# Effects of Interactions with Cats in Domestic Environment on the Psychological and Physiological State of Their Owners: Associations among Cortisol, Oxytocin, Heart Rate Variability, and Emotions

**DOI:** 10.3390/ani13132116

**Published:** 2023-06-26

**Authors:** Takumi Nagasawa, Yuichi Kimura, Koji Masuda, Hidehiko Uchiyama

**Affiliations:** 1 Department of Human and Animal-Plant Relationships, Graduate School of Agriculture, Tokyo University of Agriculture, Funako 1737, Atsugi 243-0034, Japan; cotaronagasawa@gmail.com (T.N.); k3masuda@nodai.ac.jp (K.M.); 2 Department of Animal Science, Faculty of Agriculture, Tokyo University of Agriculture, Funako 1737, Atsugi 243-0034, Japan

**Keywords:** cat ownership, human-cat interaction, oxytocin, cortisol, autonomic nervous system, emotion

## Abstract

**Simple Summary:**

In Japan, cats are popular companion animals. However, the details of the effects of direct communication with cats on the psychological and physiological states of their owners remain unknown. In this study, we conducted a remote-format experiment with 32 cat owners in their homes. Owners were requested to interact with their cats for 10 min in a routine manner. The results showed that interactions with cats decreased the emotional arousal and parasympathetic activity of the owners and increased their heart rates. Positive correlations were also noted between heart rate and cortisol concentration as well as between cortisol and oxytocin concentration. The results of this study indicate that interactions with cats at home has an excitatory effect on the physiological aspects of owners, which is in contrast with findings of previous studies that proposed stress reduction. This explains a new aspect of the mechanism of the health-promoting effects of cat ownership.

**Abstract:**

Interactions with animals, including cats, is believed to influence human health. However, studies that investigate the psychological and physiological effects of interacting with cats in their household environment are limited. In this remote study, 32 cat owners in Japan participated from June to October 2021. They completed two tasks, each on separate days in their homes: one simulating daily cat communication (Interaction condition) and another with no interactions (Rest condition). We quantified emotions (arousal level and pleasure level) before and after each condition using the Two-Dimensional Mood Scale Short-term as well as salivary cortisol and oxytocin levels of owners using enzyme-linked immune-sorbent assay. Autonomic nervous activity (sympathetic and parasympathetic) was also quantified by heart rate variability analysis. The free interaction with cats decreased emotional arousal and parasympathetic activity, and lead to increased heart rates in owners. There was a positive correlation between heart rate and cortisol concentration, and between cortisol and oxytocin concentration. Furthermore, the frequency of petting the cats was negatively correlated with the rate of change in the parasympathetic activity. In contrast, the parasympathetic nerves in the owners were activated under the Rest condition. Hence, the mechanism of health-enhancing effects of cat ownership includes an arousing effect, in contrast to the previously proposed stress-reduction effect. This result can aid in future developments in cat–human relationship studies. However, a detailed study with a larger sample size is needed to draw definite conclusions.

## 1. Introduction

In Japan, cats are as popular as dogs as pets [1]. Owners recognize cats as members of their families and live with them in a domestic environment [2]. One of the motivating factors for keeping cats include the associated health-enhancing benefits [1]. Elderly people who keep cats have a longer life expectancy than those who do not [3]. Cat ownership lowers their risk of cardiovascular diseases [4,5]. However, few studies have investigated the health benefits from cats compared to those from dogs. Moreover, the findings of these studies have been inconsistent, with some reporting positive effects, negative effects, mixed effects, or no effects of cat ownership on the health of their owners, including conditions such as cardiovascular disease, obesity, and mental well-being [6,7,8]. These inconsistent findings may be attributed to the unclear understanding of which factors, such as daily interaction and the relationship between cats and their owners, influence the health status of cat owners.

Daily direct interaction with cats may play an important role in the health-promoting effects of cat ownership. Previous studies have shown that interactions with cats increases the positive emotions of owners [9] and decreases their heart rate and blood pressure [10]. Furthermore, it was recently reported that contact with cats also increases salivary oxytocin levels in owners [11]. Oxytocin is a peptide hormone that has diverse health effects [12,13]. For example, oxytocin secretion leads to emotion-regulating functions, such as reducing anxiety and increasing well-being [14]. Thus, interactions with cats reduces psychological and physiological stress in their owners.

Two stress response pathways to external stimuli are present in the body: the hypothalamus-sympathetic-adrenal medullary (SAM) system, which regulates the functions of autonomic nervous system, and the hypothalamus-posterior pituitary-adrenal cortex (HPA) system, which is a humoral response to stress. These two functions are closely related to the oxytocin system [15,16]. Oxytocin secretion exhibits anti-stress effects, such as suppressing cortisol [17] and activating parasympathetic activity [18], and these effects reduce the risk of cardiovascular disease among the owners [12]. Furthermore, these physiological indicators associated with psychological status such as emotion [19,20]. Several previous studies on dogs and humans have simultaneously assessed multiple physiological and psychological indicators, including autonomic nervous activity, hormone levels, and emotions [21,22,23]. In contrast, few studies have focused on the effects of cat ownership on the owners, and such studies mostly used a single psychological and physiological indicator to evaluate them.

One of the major challenges in research that investigates the direct interactions between cats and their owners is the difficulty in replicating everyday interactions. Cats are territorial in nature [24], which makes it stressful for the cats if the researcher visits the homes or if the cat is moved into an experimental environment such as a laboratory. Similarly, the experimental environment, controlled by the intervention of the experimenter, can have psychological and physiological effects on owners [25]. Hence, if experiments are conducted in their own homes, the influence of experimenter will be eliminated to reproduce natural interactions, which is necessary to elucidate the mechanisms of the health-promoting effects of cats on their owners.

This study aimed to investigate how daily interactions with cats at home affect the psychological and physiological state of their owners. We measured the hormone levels (oxytocin and cortisol), autonomic nervous activity (sympathetic and parasympathetic), and emotions (pleasure and arousal emotions) of the owners. Further, we comprehensively explored the associations among these physiological and psychological indicators to elucidate the mechanisms underlying the health-promoting effects of cat ownership.

## 2. Materials and Methods

### 2.1. Ethical Statement

This study was conducted with the approval of the Human Ethics Committee (Approval No. 2001) and the Laboratory Animal Ethics Committee (Approval No. 21133) of the Tokyo University of Agriculture, as defined by the Helsinki Convention. We obtained informed consent from the owners via email and in writing.

### 2.2. Participants and Test Animals

The present study included 32 owners (Female = 26, Male = 6; Mean age = 39.31 ± 11.61, range = 14–63) in Japan that were recruited using social media. This study was conducted between June 2021 to October 2021, including 22 participants who owned between two and five cats.

### 2.3. Experimental Protocol

The owners performed two different tasks, each on a different day. In the Interaction condition, the owners were free to replicate their daily interactions with their cats for 10 min. In the Rest condition, on the contrary, the owners spent time in the space with their cats, but active interactions, such as touching or talking to them, were limited, and they spent 10 min in a resting state. To eliminate the order effect, the task day was randomized for each owner.

To avoid external interruptions, owners conducted the experiment on days when there were no visits by acquaintances or home deliveries. Further, owners conducted the experiments on days when there were no other events, such as veterinary visits, house construction, or new animal adoptions. All experimental work was carried out by the owners themselves, while alone, to eliminate any impact on the cats caused by the visits of experimenters. On the day before the experimental day, we mailed the equipment and questionnaires necessary for the experiment to the owners’ homes. In addition, the owners were familiarized with the flow of the experiment and the use of the tools through paper and video materials prior to the experiment.

**Figure 1:**
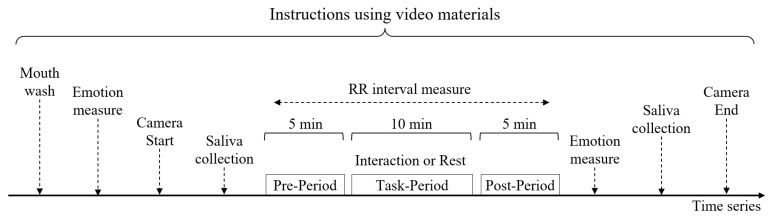
Experimental protocol flow.

On the day of the experiment, the owners followed the image and audio instructions on the video material prepared in advance by the experimenter (Figure 1). First, the owners cleaned their mouths and completed a questionnaire to measure their emotions within approximately 5 min. The owner then started recording the camera and performed the first saliva collection. The collected saliva was promptly stored at −20 °C within approximately 5 min. The R-R wave intervals of the owners were then measured using a PolarV800 for a total of 20 min (5 min Pre, 10 min Task, 5 min Post). During the Pre and Post times, the owner remained at rest, either in a chair or on the ground. The owners then responded to a second emotional questionnaire, saliva was collected, and the camera recording was stopped.

### 2.4. Hormones Assay Methods

#### 2.4.1. Collection of Saliva Samples

To ensure the reliability of hormone concentrations, the participants did not perform any dental procedures on the day of or the day before the experiment. In addition, all experiments were conducted between 12:00 and 17:00 to eliminate the effects of diurnal variations. Saliva samples were collected using the drooling method. The participants were asked to rinse their mouths with water to remove impurities within the 10 min prior to saliva collection. Participants transferred saliva into a dedicated 2 mL tube (Cryovial, 2 mL, White, SalivaBio, 5004.01, Salimetrics LLC., State College, PA, USA) using a dedicated syringe (Saliva Collection Aid, 5016.02, Salimetrics LLC., State College, PA, USA) in the mouth and aimed to collect at least 1.5 mL of saliva, which was transferred into the tubes. Thereafter, the samples were promptly frozen and stored in a −20 °C freezer at home. Consequently, these samples were delivered to the laboratory and stored at −20 °C until hormone concentrations were measured. Before measuring hormone concentrations, the samples were thawed and centrifuged at 1087× *g* for 15 min at 4 °C, and the supernatant fluid was extracted.

#### 2.4.2. Oxytocin

The concentration of oxytocin in the saliva was measured as previously described [26]. Participants’ saliva samples (250–1000 μL) were dried in a centrifugal concentrator, reconstituted to 250 μL using Assay Buffer, and used for oxytocin quantification. Oxytocin concentrations were quantified using an ENZO oxytocin enzyme immunoassay kit (ADI-901-153, ENZO Life Sciences, Inc., Farmingdale, NY, USA). Concentrations were determined based on the volume of saliva collected from each participant. The standard curve ranged from 15.6–1000 pg/mL with a sensitivity of 15.0 pg/mL. Absorbance measurements were performed on an iMark microplate reader (Bio-Rad Laboratories, Inc., Tokyo, Japan), corrected at 590 nm and measured at 405 nm; intra-assay CV was 2.00%, and inter-assay CV was 7.04%.

#### 2.4.3. Cortisol

Saliva samples were diluted 5-fold with assay buffer to prevent matrix interference and to ensure that the calculated concentrations were within the standard curve. The ENZO cortisol enzyme immunoassay kit (ADI-900-071, ENZO Life Sciences, Inc., Farmingdale, NY, USA) was used to determine the concentration of cortisol in saliva. The standard curve ranged from 156 to 10,000 pg/mL with a sensitivity of 56.72 pg/mL. Absorbance measurements were performed using an iMark microplate reader (Bio-Rad, Tokyo, Japan), corrected at 590 nm, and measured at 405 nm. The intra-assay CV and inter-assay CV were 2.12% and 9.47%, respectively.

### 2.5. Autonomic Nervous System

Heart rate (HR) and R-R wave intervals (RR) were measured using a Polar V800 (Polar Electro Japan, Tokyo, Japan). A belt with a sensor attached was noninvasively placed on the participant’s chest. Heart rate variability analysis was performed on the obtained R-R wave interval data using Kubios HRV Software 3.4.2 (Kubios Oy, Kuopio, Finland). The automatic correction feature was applied to remove artifacts in the data [27]. Consequently, standard deviation of normal-to-normal intervals (SDNN), root mean square successive difference (RMSSD), high frequency (HF), and low frequency (LF) were calculated. Parasympathetic activity was assessed using RMSSD and HF, while sympathetic activity was assessed using SDNN divided by RMSSD and LF divided by HF.

### 2.6. Questionnaires

The Two-Dimensional Mood Scale Short-term (TDMS-ST) was used to measure the emotions of the owners [28]. The vitality and stability levels of the owners were quantified between −10 and 10. Further, pleasure level, the sum of vitality and stability, and the arousal level, vitality minus stability were quantified between −20 and 20. The attachment level of the owners to their cats was quantified using the Lexington Attachment to Pets Scale (LAPS) [29].

### 2.7. Behavioral Analyses

An action camera (MUSON MAX1, soundpeatsaudio Ltd., Shenzhen, China) was used to capture the interaction of the owner with the cat during the experiment. The owner used a camera attached to a head-mounted strap. The categories and definitions of the behaviors to be analyzed are listed in Table 1. The analysis method employed a 1-0 sampling method with 5-s intervals [30].

### 2.8. Statistical Analyses

All statistical analyses were performed using Bell Curve for Excel (Social Information Service, Tokyo, Japan). Statistical significance was set at *p* < 0.05. We used Wilcoxon signed-rank test to identify variations before and after Task in oxytocin, cortisol, and emotion and to calculate effect sizes (r). We also used the Friedman test and Scheffe’s method to ascertain the variability among Pre, Task, and Post in autonomic nervous activity and calculated effect sizes (r).

We calculated the percentage change in hormone concentrations and autonomic nervous activity from Pre to Post. For emotional values, we calculated the amount of change by subtracting Pre from Post. Using Spearman’s rank correlation coefficient, we confirmed the association between these psychological and physiological variables.

We calculated principal component scores by applying principal component analysis (PCA) to the frequency of owner interactions with cats using video data. Rather than revealing the potential principal component structure, we aimed to reduce variables to create an overall synthetic variable. Therefore, PCA was performed without rotation [31]. Spearman’s rank correlation coefficient was used to ascertain whether principal component scores were associated with psychological and physiological variation in participants.

## 3. Results

Participants who declined to perform any experiment (Interaction condition: one; Rest condition: two) and those who did not follow the experimental protocol (Interaction condition: five; Rest condition: six) were excluded from all analyses. In addition, participants with incomplete oxytocin (Interaction condition: three; Rest condition: two three), cortisol (Interaction condition: two; Rest condition: one), RR interval data (Interaction condition: four; Rest condition: three), attachment score (one), and video footage (three) were excluded from the individual analyses. Supplemental Material Table S1 contains information on all analysis datasets.

### 3.1. Interaction Condition

#### 3.1.1. Variation in Psychological and Physiological Indicators

The results of variations in different indicators are shown in Table 2 and Table 3. After Task, arousal level decreased. During Task, RR and HF decreased significantly, whereas HR increased significantly.

#### 3.1.2. Relevance of Psychological and Physiological Indicators

Table 4 shows the correlation coefficients between different psychological and physiological indicators. Oxytocin was positively correlated with arousal (r = 0.43). Cortisol correlated positively with HR (r = 0.45) and oxytocin (r = 0.51) and negatively with RR (r = −0.45).

#### 3.1.3. Type and Frequency of Interactions

Principal component analysis was performed on the frequency of interactions between owners and their cats. The criteria for the principal components were an eigenvalue > 1 and a cumulative contribution > 70%. Three principal components (PC1, PC2, PC3, and Table 5) were extracted.

Table 6 represents the results of correlation analysis between psycho-physiological indicators and principal component scores. PC2 was positively correlated with SDNN (r = 0.46), RMSSD (r = 0.48), and LF (r = 0.49).

#### 3.1.4. Age and LAPS of Owners

The results of correlation analysis between owner age, ownership experiment, and LAPS are shown in Table 7. LAPS was positively correlated with pleasure level (r = 0.48).

### 3.2. Rest Condition

#### 3.2.1. Variation in Psychological and Physiological Indicators

The results of variations in psychological and physiological indicators are presented in Table 8 and Table 9. After Task, the vitality, pleasure, and arousal levels of the participants decreased significantly, whereas RMSSD and HF increased significantly during Task.

#### 3.2.2. Relevance of Psychological and Physiological Indicators

The correlation coefficients are shown in Table 10. Stability was negatively correlated with the LF/HF ratio (r = −0.47). Oxytocin was positively correlated with RMSSD (r = 0.51), HF (r = 0.52), and cortisol (r = 0.52).

#### 3.2.3. Age and LAPS of Owners

The results of correlation analysis between owner age, ownership experiment, and LAPS are shown in Table 11. There was no significant correlation.

## 4. Discussion

The results of the present study indicate that interactions with cats affect multiple psychological and physiological indicators of their owners. In this study, we observed activation of sympathetic nervous activity in cat owners, including an increase in heart rate, which is inconsistent with the results of a previous study [10]. Conventionally, the health-enhancing effects of animal interactions have been attributed to the stress-reducing effects or calming effects of such interactions on one’s psychological and physiological state [32,33]. Most previous studies that investigated animal-mediated interventions were conducted with individuals who were already suffering from some disease (e.g., mental health) [32,33,34,35]. They were under chronic high stress owing to disease symptoms, hence stress reduction effects of animals play an important role as an adjunct to their treatment [32,33]. Although dogs and horses have been utilized more frequently in therapeutic studies [34], cats have also used for therapeutic activities for patients with diverse diseases [34,36]. However, it may be inappropriate if the same health-promoting mechanisms of therapy animals were assumed for healthy owners. In the present study, we established a completely remote protocol that allowed owners to carry out all the experiments themselves in their most relaxed home environment, thus eliminating the intervention of an external researcher. Hence, this study replicated more natural interaction situations in the daily life of the owners. The results indicated that interactions with cats might place the autonomic nervous activity of the owners in an arousal-like state. That is, the results suggest that stress reduction, despite being an important aspect of the health-promoting effects of animals, was not observed during pet interactions in a safe and relaxed home environment.

Stimuli that produce a stress response in an organism are referred to as stressors, which can produce two types of stress responses: distressed and eustressed [37]. A eustress response is a psycho-physiological response brought about by a positive stressor for the stimulus recipient that can lead to increased vitality and health of a person [37,38]. The eustress response can potentially promote physical resilience and resistance to diseases under exposure to moderate stress within its own controllable range and may produce physical and mental health benefits [39,40,41]. The results of this study suggest that interactions with cats can trigger a eustress response in the mind and body of the owner as a moderate stimulus for the owner.

In the present study, oxytocin levels in most participants were elevated after interactions with cats, but the variation was not statistically significant. This result is consistent with those of a previous study [11]. In contrast, the rate of change in cortisol was positively correlated with the rate of change in oxytocin and heart rate of the owners. In several previous studies, it has been reported that salivary oxytocin and cortisol levels both increased after tasks that induce stress [42,43] and cortisol-induced oxytocin secretion is also believed to be a part of the mechanism of stress processing [44]. Cortisol concentration in saliva and the variability of heart rate are meaningful biomarkers for assessing physiological and psychological stress as well as arousal states in humans [45,46]. Thus, the results of the present study suggest the existence of a physiological mechanism by which the stimuli of interactions with cats arouse the physiological state of their owners, thereby increasing oxytocin levels. In contrast, certain previous studies on the health-enhancing effects of cats on their owners have focused on psychological emotions [9], blood pressure, heart rate [10], and oxytocin systems [11]. The present study focused on multiple psychological and physiological indicators, and the results suggest that fluctuations in the HPA are interrelated with those in SAM systems due to interactions with cats. That is, the results highlighted the significance of assessing multiple rather than single indicators to investigate the health-promoting effects of cat ownership.

However, the relationship between the HPA system, SAM system, and psychological aspects is highly complex. The relationship between cortisol and oxytocin remains debated and complex [15,17]. The results of the present study also showed that the emotional arousal of the owners decreased significantly after interactions with cats. This is in contrast to changes in autonomic nervous activity, such as an increase in heart rate after the interaction. Further, a positive correlation exists between the amount of change in arousal level and oxytocin concentration. However, the reasons for this complex relationship are difficult to interpret. For example, one hypothesis suggests that this is due to a subjective bias in the assessment of psychological aspects. Participants in the present study were aware that the primary impact of animals on people was physical relaxation, and this cognitive bias may have influenced their psychological responses. The gap in the timing between the psychological and physiological assessments may also have influenced the results [47]. Autonomic nervous activity during interactions with the cats was assessed in real time, whereas emotional evaluations were assessed after a slight delay, following the interaction. In this study, emotion was measured after the 5 min Post period, rather than immediately after the 10 min Task period. Participants may have been relieved from a sympathetic upswing in autonomic nervous activity, and the reaction may have resulted in more psychological arousal reduction. However, these interpretations are only predictions. While the evaluation of multiple psychological and physiological measures is important in investigating the health-enhancing effects of cats, it should be performed with caution.

In the present study, PCA was also used to calculate the composite variables of multiple interactions performed on cats. The results showed that petting behaviors had a negative effect, whereas playing behaviors had a positive effect on the rate of parasympathetic change, such as the RMSSD of the owners. Interactions, such as petting and playing, are common behaviors for many owners, hence their employment as question items in scales that quantify owner-cat relationships [48]. The results of this study suggest that the autonomic nervous activity of many cat owners fluctuates with their daily interactions with their cats.

RMSSD is a measure of parasympathetic activity and forms a negative correlation with sympathetic nervous system activity, such as heart rate. Thus, the behavior of playing with the cat may increase the parasympathetic nervous system of the owner, whereas the behavior of petting the cat may activate the sympathetic nervous system. In this study, all owners engaged in petting their cats during the 10 min interaction, indicating that this type of interaction is significant for owners (Supplementary Materials Table S1). Contact stimulation is known to have a healing effect, causing a decrease in heart rate and cortisol levels [49], which contrasts with the results of the present study. One reason for this is that the owners were allowed to interact freely with their cats. In order to replicate the typical cat–owner interaction scenarios within each household and meet the objectives of the study, we did not impose restrictions on how owners petted their cats in terms of location, speed, or intensity. Owners may experience increased sympathetic activity as a result of petting actively rather than slow petting of the cat for relaxation. Interpreting the results remains speculative. Furthermore, it should be noted that the owners used a cat toy to play with their cats, preventing them from petting the cats during that time. This contrast in interaction may have contributed to a predominance of parasympathetic activity in owners who engaged in play with their cats. However, since not all owners engaged in playful behavior (Supplementary Materials), a definitive interpretation cannot be made.

In this study, interactions involving petting the cat may have likely influenced the increase in oxytocin levels of the owner, which is consistent with the results of a previous study [11]. Contact stimuli have been shown to increase oxytocin levels [49], which is also consistent with the present study. Oxytocin contributes to improved psychological and physiological health [12,14], and it likely plays a part in the physiological mechanisms of health effects in human–animal relationships [50]. In essence, there may be a health-promoting mechanism whereby communication through contact with cats in a safe and relaxing home environment offers moderate physiological stimulation and triggers oxytocin secretion. However, as mentioned earlier, owner–cat interactions are complex and cannot be easily categorized into a single distinct behavior. In this study, we could only consider the overall influence of cat interactions on the physiological aspects of their owners.

In the Rest condition, the parasympathetic activities of the owners increased, whereas vitality and pleasure levels decreased. A positive correlation was found between parasympathetic activity and oxytocin, and a negative correlation between sympathetic activity and stability level. These results contradicted our expectation that the psychological and physiological states of the owners are not changed. In the control condition of the previous study, participants read a book [11]. In the present study, the control condition was to spend time doing nothing to account for the presence of owners for whom reading was not a daily habit. However, the diversity of activities in the houses of participants, and the frequency of interactions with cats may have influenced the results of the control condition. In future, the control condition should be carefully set, and a more neutral set of conditions is required.

This study had a few limitations. First, it was difficult to strictly control the experimental conditions. To set up natural communication situations between owners and cats, this study employed a fully remote format experimental protocol using video materials. The protocol of this study was beneficial for controlling unwanted influences on cats and owners. However, the home environments of participants (e.g., house size and number of furniture) were diverse, as opposed to a common environment such as a laboratory. This could serve as a bias when making comparisons between owners. Moreover, in this study, the owners were free to change their posture (standing or sitting) and move during the 10 min task. In other words, it is possible that physical activity other than direct interaction with the cat also affected the physiological fluctuations of the owners. However, because it was difficult to strictly distinguish between interaction and other physical activity, no corresponding analysis was performed.

Second, it is possible that the protocol of this experiment itself induced a physiological stress response in the owners. To minimize this effect as much as possible, we instructed owners to practice operating the experimental equipment and created a protocol that could be performed in their home. However, because this study was not a naturalistic observation, the effects of the experiment cannot be completely eliminated. In future studies, efforts should be made to eliminate any unwanted effects of the experiment itself.

In addition, owing to the lack of experimenter intervention, several owners were unable to accurately carry out the experimental protocol or sample saliva or RR interval data. These data could not be used in the analysis, resulting in a reduced number of samples for analysis. Due to the small sample size, we have not observed a clear association between variables. A study with a larger sample size should be conducted.

Furthermore, owner and cat demographic attributes are factors that influence the quantity and quality of their interactions, but were not analyzed in depth in this study. For example, compared to male owners, female owners may be more likely to have smoother interactions and better relationships with their cats [51,52]. One possible reason for this could be the tendency of women to vocalize for cats [53] and to be at the same height as the cat during interactions [54]. Previous reports have indicated that women, particularly those with neurotic tendencies, are more likely to exhibit increased activity in the prefrontal cortex during interactions with cats [55]. Although the proportion of women was high in this study, the sample size was too small to analyze gender differences. Additionally, the cat’s personality (gender [56], characteristic temperament [57], and type of attachment toward the owner [58]) may also present a factor affecting the quality and quantity of cat–owner interactions. However, this study was not able to analyze the results for owners living with multiple cats, taking into account their relationships and interactions with individual cats. Therefore, future studies should focus on these factors as well.

## 5. Conclusions

The present study showed that interactions with cats may improve the physiological state of their owners. Traditionally, interactions with pets, including cats, have been proposed to have a relaxing effect on people. The results of the present proposed a new mechanism, i.e., an arousal system, for the health-enhancing effects of cat ownership.

In addition, communication, especially through contact, was found to be a stimulant for the activation of the owner’s the sympathetic nervous system. There was also a positive correlation between heart rate and cortisol concentration, and between cortisol and oxytocin concentration, suggesting that interactions with cats at home can even influence the oxytocin system. These results, obtained through experiments that observed daily interaction situations between cats and their owners in the domestic environment, which have not been conducted before, can aid in future developments in cat–human relationship studies. However, due to the small sample size and gender bias of this study, no clear conclusions can be drawn, and more detailed research is needed.

## Figures and Tables

**Table 1:** List of actions and categories to be analyzed.

Categories	Definition
Petting	Pet or stroke a cat (e.g., its face and body)
Hugging	Pick up or hold a cat
Brushing	Brush a cat with a brush or comb
Feeding	Feed a cat (food or treats)
Playing	Invite a cat to play using a cat toy
Calling	Call the cat’s name
Talking	Talking with a cat (e.g., greeting and baby talk)

**Table 2:** Results of statistical analysis in emotions and hormones in the Interaction condition.

Indicators		*n*	Pre (Mean ± SD)	Post (Mean ± SD)	Wilcoxon Signed-Rank Test				
					Increased (*n*)	No change (*n*)	Decreased (*n*)	*p*-Value	Effect Value (r)
Emotion	Vitality level	26	1.46 ± 4.17	0.27 ± 4.98	10	2	14	0.096	0.33
	Stability level	26	6.92 ± 2.56	7.77 ± 2.58	17	2	7	0.081	0.34
	Pleasure level	26	8.38 ± 5.26	8.04 ± 4.89	10	3	13	0.572	0.11
	Arousal level	26	−5.46 ± 4.49	−7.5 ± 6.24	5	2	19	**0.013**	0.49
Hormone	Oxytocin (pg/mL)	23	104.34 ± 158.45	94.84 ± 132.43	15	0	8	0.362	0.19
	Cortisol (ng/mL)	24	3.46 ± 4.91	3.94 ± 7.30	12	0	12	0.864	0.03

The mean ± SD for Pre and Post are shown. The results of the Wilcoxon signed-rank test are shown; in the *p*-value column, numbers below 0.05 are bolded.

**Table 3:** Results of statistical analysis in autonomic nervous activity in the Interaction condition.

Indicators	Pre	Task	Post	Friedman Test and Scheffe’s Method			
				Items	Chi-Square Value	*p*-Value	Effect Value (r)
RR (ms)				Friedmann Test	25.182	**<0.001**	1.07
(Mean ± SD)	827.23 ± 101.48	768.14 ± 88.78	834.51 ± 115.64	Pre vs. Task	15.364	**<0.001**	0.84
(Mean Rank)	2.320	1.140	2.550	Pre vs. Post	0.568	0.753	0.16
				Task vs. Post	21.841	**<0.001**	1.00
HR (bpm)				Friedmann Test	25.182	**<0.001**	1.07
(Mean ± SD)	73.52 ± 8.49	79.17 ± 9.01	73.11 ± 9.28	Pre vs. Task	15.364	**<0.001**	0.84
(Mean Rank)	1.682	2.864	1.455	Pre vs. Post	0.568	0.753	0.16
				Task vs. Post	21.841	**<0.001**	1.00
SDNN (ms)				Friedmann Test	0.636	0.728	0.17
(Mean ± SD)	35.61 ± 32.91	32.98 ± 24.37	30.1 ± 14.14	Pre vs. Task	0.568	0.753	0.16
(Mean Rank)	1.909	2.136	1.955	Pre vs. Post	0.023	0.989	0.03
				Task vs. Post	0.364	0.834	0.13
RMSSD (ms)				Friedmann Test	0.091	0.956	0.06
(Mean ± SD)	30.56 ± 31.34	28.01 ± 25.27	24.88 ± 13.97	Pre vs. Task	0.091	0.956	0.06
(Mean Rank)	2.046	1.955	2.000	Pre vs. Post	0.023	0.989	0.03
				Task vs. Post	0.023	0.989	0.03
SDNN/RMSSD				Friedmann Test	1.091	0.580	0.22
(Mean ± SD)	1.23 ± 0.23	1.30 ± 0.28	1.30 ± 0.37	Pre vs. Task	0.818	0.664	0.19
(Mean Rank)	1.818	2.091	2.091	Pre vs. Post	0.818	0.664	0.19
				Task vs. Post	0.000	1.000	<0.001
LF (ms^2^)				Friedmann Test	1.909	0.385	0.29
(Mean ± SD)	744.69 ± 891.68	934.93 ± 2345.12	665.44 ± 726.64	Pre vs. Task	0.205	0.903	0.10
(Mean Rank)	2.182	2.046	1.773	Pre vs. Post	1.841	0.398	0.29
				Task vs. Post	0.818	0.664	0.19
HF (ms^2^)				Friedmann Test	8.273	**0.016**	0.61
(Mean ± SD)	316.48 ± 336.35	514.23 ± 1580.91	338.61 ± 461.05	Pre vs. Task	8.205	**0.017**	0.61
(Mean Rank)	2.409	1.546	2.046	Pre vs. Post	1.455	0.483	0.26
				Task vs. Post	2.750	0.253	0.35
LF/HF				Friedmann Test	4.455	0.108	0.45
(Mean ± SD)	2.84 ± 2.23	3.51 ± 2.43	5.08 ± 11.51	Pre vs. Task	2.750	0.253	0.35
(Mean Rank)	1.864	2.364	1.773	Pre vs. Post	0.091	0.956	0.06
				Task vs. Post	3.841	0.147	0.42

The mean ± SD and mean ranks for Pre, Task, and Post are shown. The results of the Friedman test and Scheffe’s method are shown; in the *p*-value column, numbers below 0.05 are bolded.

**Table 4:** Result of correlation analysis between psychological and physiological indicators in the Interaction condition.

		1	2	3	4	5	6	7	8	9	10	11	12	13	14
Vitality level	1	1.00													
Stability level	2	−0.06	1.00												
Pleasure level	3	0.65 **	0.61 **	1.00											
Arousal level	4	0.78 **	−0.59 **	0.15	1.00										
RR (ms)	5	−0.19	−0.04	−0.15	−0.21	1.00									
HR (bpm)	6	0.19	0.04	0.15	0.21	−1.00	1.00								
SDNN (ms)	7	0.01	−0.26	−0.03	0.13	0.00	0.00	1.00							
RMSSD (ms)	8	−0.07	0.02	0.09	−0.08	0.53 *	−0.53 *	0.53 *	1.00						
SDNN/RMSSD	9	0.13	−0.28	−0.10	0.26	−0.53 *	0.53 *	0.15	−0.72 **	1.00					
LF (ms^2^)	10	0.23	−0.01	0.23	0.17	−0.16	0.16	0.75 **	0.17	0.44 *	1.00				
HF (ms^2^)	11	−0.18	−0.01	0.00	−0.18	0.57 **	−0.57 *	0.33	0.89 **	−0.72 **	−0.05	1.00			
LF/HF	12	0.38	−0.07	0.23	0.34	−0.50 *	0.50 *	0.31	−0.47 *	0.81 **	0.71 **	0.66 **	1.00		
Oxytocin (pg/mL)	13	0.26	−0.36	0.19	0.43 *	−0.29	0.29	0.10	0.00	0.08	0.12	−0.04	0.22	1.00	
Cortisol (ng/mL)	14	0.12	−0.39	−0.08	0.25	−0.45 *	0.45 *	0.19	0.04	0.09	0.06	0.14	0.04	0.51 *	1.00

The numbers in the table show Spearman’s correlation coefficients. *p* values smaller than 0.05 are shown in bold. * *p* < 0.05, ** *p* < 0.01.

**Table 5:** Results of principal component analysis for interactions between owners and cats.

Interaction Items	PC1	PC2	PC3
Talking	**0.89**	0.06	0.11
Calling	**0.88**	0.09	0.02
Playing	−0.27	**0.87**	0.02
Petting	−0.17	**−0.85**	0.31
Hugging	−0.24	−0.16	**−0.88**
Feeding	**0.76**	0.09	−0.08
Brushing	**0.57**	−0.26	−0.38
Eigenvalue	2.64	1.59	1.04
Contribution rate (%)	37.67	22.73	14.89
Cumulative contribution rate (%)	75.29		

Loads in excess of 0.05 are shown in bold.

**Table 6:** Result of correlation analysis between psycho-physiological indicators and principal component scores.

Indicators		PC1	PC2	PC3
Emotion	Vitality level	0.27	0.12	−0.27
	Stability level	0.16	0.08	−0.09
	Pleasure level	0.36	0.19	−0.26
	Arousal level	0.16	0.05	−0.17
Autonomic nervous activity	RR (ms)	0.26	−0.04	0.15
	HR (bpm)	−0.26	0.04	−0.15
	SDNN (ms)	−0.31	0.46 *	0.03
	RMSSD (ms)	0.19	0.48 *	0.12
	SDNN/RMSSD	−0.42	−0.22	−0.07
	LF (ms^2^)	−0.37	0.49 *	−0.12
	HF (ms^2^)	0.26	0.37	0.10
	LF/HF	−0.29	0.16	−0.16
Hormone	Oxytocin (pg/mL)	0.00	−0.02	0.12
	Cortisol (ng/mL)	−0.16	0.17	0.01

The numbers in the table show Spearman’s correlation coefficients. * *p* < 0.05. *p* values smaller than 0.05 are shown in bold.

**Table 7:** Correlations between owner age and attachment to psycho-physiological indicators and principal component scores.

Indicators		Age	LAPS
Emotion	Vitality level	0.02	0.28
	Stability level	−0.28	0.27
	Pleasure level	−0.13	0.48 *
	Arousal level	0.31	−0.02
Autonomic nervous activity	RR (ms)	0.11	−0.23
	HR (bpm)	−0.11	0.23
	SDNN (ms)	0.07	−0.21
	RMSSD (ms)	0.20	−0.25
	SDNN/RMSSD	−0.07	0.09
	LF (ms^2^)	0.00	−0.07
	HF (ms^2^)	0.07	−0.26
	LF/HF	0.03	0.09
Hormone	Oxytocin (pg/mL)	0.14	0.08
	Cortisol (ng/mL)	−0.20	0.12
Interaction	PC1	−0.20	0.13
	PC2	−0.01	−0.21
	PC3	−0.08	0.09

The numbers in the table show Spearman’s correlation coefficients. * *p* < 0.05.

**Table 8:** Results of statistical analysis in emotions and hormones in the Rest condition.

Indicators		*n*	Pre (Mean ± SD)	Post (Mean ± SD)	Wilcoxon Signed-Rank Test				
					Increased (*n*)	No Change (*n*)	Decreased (*n*)	*p*-Value	Effect Value (r)
Emotion	Vitality level	24	2.04 ± 3.76	−0.96 ± 4.14	2	3	19	**<0.001**	0.73
	Stability level	24	6.46 ± 1.96	7.04 ± 2.40	13	8	3	0.122	0.32
	Pleasure level	24	8.50 ± 3.97	6.08 ± 3.99	3	4	17	**0.004**	0.60
	Arousal level	24	−4.42 ± 4.49	−8.00 ± 5.48	4	3	17	**<0.001**	0.73
Hormone	Oxytocin (pg/mL)	21	124.56 ± 161.47	137.01 ± 197.60	14	0	7	0.085	0.38
	Cortisol (ng/mL)	23	3.30 ± 3.83	3.62 ± 4.66	10	0	13	0.808	0.05

The mean ± SD for Pre and Post are shown. The results of the Wilcoxon signed-rank test are shown; in the *p*-value column, numbers below 0.05 are bolded.

**Table 9:** Results of statistical analysis in autonomic nervous activity in the Rest condition.

Indicators	Pre	Task	Post	Friedmann Test and Scheffe’s Method			
				Items	Chi-Square Value	*p*-Value	Effect Value (r)
RR (ms)				Friedmann Test	2.571	0.277	0.35
(Mean ± SD)	823.66 ± 113.28	835.61 ± 117.74	837.84 ± 108.93	Pre vs. Task	1.929	0.381	0.30
(Mean Rank)	1.714	2.143	2.143	Pre vs. Post	1.929	0.381	0.30
				Task vs. Post	0.000	1.000	<0.001
HR (bpm)				Friedmann Test	2.571	0.277	0.35
(Mean ± SD)	74.15 ± 10.02	73.23 ± 10.49	72.82 ± 9.94	Pre vs. Task	1.929	0.381	0.30
(Mean Rank)	2.286	1.857	1.857	Pre vs. Post	1.929	0.381	0.30
				Task vs. Post	0.000	1.000	<0.001
SDNN (ms)				Friedmann Test	2.381	0.304	0.34
(Mean ± SD)	25.02 ± 12.6	27.02 ± 10.96	26.91 ± 13.9	Pre vs. Task	0.595	0.743	0.17
(Mean Rank)	1.762	2.000	2.238	Pre vs. Post	2.381	0.304	0.34
				Task vs. Post	0.595	0.743	0.17
RMSSD (ms)				Friedmann Test	7.238	**0.027**	0.59
(Mean ± SD)	20.48 ± 10.3	25.69 ± 13.64	25.05 ± 13.61	Pre vs. Task	6.095	**0.048**	0.54
(Mean Rank)	1.524	2.286	2.191	Pre vs. Post	4.667	0.097	0.47
				Task vs. Post	0.095	0.954	0.07
SDNN/RMSSD				Friedmann Test	4.571	0.102	0.47
(Mean ± SD)	1.27 ± 0.32	1.14 ± 0.34	1.12 ± 0.24	Pre vs. Task	3.429	0.180	0.40
(Mean Rank)	2.381	1.810	1.810	Pre vs. Post	3.429	0.180	0.40
				Task vs. Post	0.000	1.000	<0.001
LF (ms^2^)				Friedmann Test	1.238	0.539	0.24
(Mean ± SD)	456.32 ± 756	339.6 ± 340.38	389.99 ± 486.52	Pre vs. Task	0.095	0.954	0.07
(Mean Rank)	2.143	2.048	1.810	Pre vs. Post	1.167	0.558	0.24
				Task vs. Post	0.595	0.743	0.17
HF (ms^2^)				Friedmann Test	10.571	**0.005**	0.71
(Mean ± SD)	229.42 ± 317.63	382.59 ± 457.57	303.24 ± 386.73	Pre vs. Task	10.500	**0.005**	0.71
(Mean Rank)	1.476	2.476	2.048	Pre vs. Post	3.429	0.180	0.40
				Task vs. Post	1.929	0.381	0.30
LF/HF				Friedmann Test	4.952	0.084	0.49
(Mean ± SD)	3.1 ± 3.59	1.92 ± 2.16	1.83 ± 1.87	Pre vs. Task	4.667	0.097	0.47
(Mean Rank)	2.381	1.714	1.905	Pre vs. Post	2.381	0.304	0.34
				Task vs. Post	0.381	0.827	0.13

The mean ± SD and mean ranks for Pre, Task, and Post are shown. The results of the Friedman test and the Scheffe’s method are shown; in the *p*-value column, numbers below 0.05 are bolded.

**Table 10:** Result of correlation analysis between psychological and physiological indicators in the Rest condition.

		1	2	3	4	5	6	7	8	9	10	11	12	13	14
Vitality level	1	1.00													
Stability level	2	−0.17	1.00												
Pleasure level	3	0.74 **	0.46 *	1.00											
Arousal level	4	0.80 **	−0.60 **	0.29	1.00										
RR (ms)	5	0.11	0.06	0.00	0.12	1.00									
HR (bpm)	6	−0.11	−0.06	0.00	−0.12	−1.00	1.00								
SDNN (ms)	7	−0.11	−0.12	−0.14	0.05	0.13	−0.13	1.00							
RMSSD (ms)	8	−0.01	−0.12	−0.11	0.13	0.36	−0.36	0.77 **	1.00						
SDNN/RMSSD	9	−0.16	−0.10	−0.07	−0.09	−0.32	0.32	0.52 *	−0.05	1.00					
LF (ms^2^)	10	−0.07	−0.43	−0.30	0.16	−0.19	0.19	0.72 **	0.45 *	0.62 **	1.00				
HF (ms^2^)	11	−0.11	0.15	−0.01	−0.11	0.28	−0.28	0.41	0.71 **	−0.26	0.14	1.00			
LF/HF	12	−0.01	−0.47 *	−0.29	0.18	−0.34	0.34	0.30	−0.12	0.71 **	0.71 **	−0.57 **	1.00		
Oxytocin (pg/mL)	13	0.06	−0.15	−0.10	0.06	0.32	−0.32	0.05	0.51 *	−0.35	0.12	0.52 *	−0.20	1.00	
Cortisol (ng/mL)	14	0.26	−0.33	0.00	0.35	0.38	−0.38	0.06	0.24	−0.11	0.22	0.27	−0.02	0.52 *	1.00

The numbers in the table show Spearman’s correlation coefficients. *p* values smaller than 0.05 are shown in bold. * *p* < 0.05, ** *p* < 0.01.

**Table 11:** Correlations between owner age and attachment to psychological and physiological indicators.

Indicators		Age	LAPS
Emotion	Vitality level	0.16	−0.06
	Stability level	−0.02	−0.15
	Pleasure level	0.15	−0.20
	Arousal level	0.26	0.00
Autonomic nervous activity	RR (ms)	0.32	0.21
	HR (bpm)	−0.32	−0.21
	SDNN (ms)	0.24	0.25
	RMSSD (ms)	0.18	0.35
	SDNN/RMSSD	0.04	−0.21
	LF (ms^2^)	−0.03	0.22
	HF (ms^2^)	0.00	0.17
	LF/HF	0.01	0.03
Hormone	Oxytocin (pg/mL)	−0.15	0.14
	Cortisol (ng/mL)	−0.06	0.28

The numbers in the table show Spearman’s correlation coefficients.

## Data Availability

The data presented in this study are available in Supplementary Materials.

## Supplementary Materials

The following supporting information can be downloaded at: https://www.mdpi.com/article/10.3390/ani13132116/s1. Table S1: Dataset.
